# Diagnostic Accuracy of Urine and Vaginal Self-Sampling for Detection of High-Risk Human Papillomavirus: A Systematic Review and Meta-Analysis

**DOI:** 10.3390/v18060681

**Published:** 2026-06-18

**Authors:** Altynshash Rakhat, Gulzhanat Aimagambetova

**Affiliations:** 1 School of Medicine, Nazarbayev University, 010000 Astana, Kazakhstan; altynshash.rakhat@nu.edu.kz; 2 Department of Surgery, School of Medicine, Nazarbayev University, 010000 Astana, Kazakhstan; 3 Clinical Academic Department of Women’s Health, Corporate Fund University Medical Center, 010000 Astana, Kazakhstan

**Keywords:** cervical cancer, human papillomavirus, HPV self-sampling, vaginal self-sampling, urine self-sampling, cervical cancer screening, diagnostic accuracy, meta-analysis

## Abstract

Cervical cancer remains a major public health challenge, particularly in low- and middle-income countries. The primary cause of cervical cancer is high-risk human papillomavirus (HPV), and screening using physician-collected samples is complicated by stigma, inconvenience, and access. There are non-invasive alternatives to the physician-collected samples, including self-sampling methods such as first-void urine and vaginal swabs. This systematic review and meta-analysis evaluated and compared the diagnostic accuracy of vaginal and urine self-sample methods for detecting high-risk HPV. PubMed, Scopus, Web of Science, and the Cochrane Library were searched for studies published between January 2015 and October 2025. Bivariate random-effects models and HSROC models were used to estimate pooled sensitivity and specificity results compared with clinician-collected samples for CIN2+. Meta-regression assessed sources of heterogeneity. Twenty-two studies involving over 9000 participants were included. Vaginal self-sampling showed a pooled sensitivity of 91.3% and a specificity of 86.9%, while urine self-sampling showed 86.9% sensitivity and 79.5% specificity. Vaginal swabs demonstrated higher sensitivity in head-to-head comparisons. DNA-based PCR assays showed higher sensitivity than mRNA-based tests, and room-temperature storage decreased urine sample sensitivity. Both methods are effective for high-risk HPV detection. Vaginal self-sampling showed superior performance, while urine self-sampling remains a valuable non-invasive option for under-screened populations.

## 1. Introduction

Cervical cancer is the second most common cancer in women in low-income countries and the fourth most common cancer in women globally, with an estimated 703,000 new cases in 2025, highlighting an important public health concern [1]. The World Health Organization (WHO) [1] reported that about 94% of cervical cancer deaths, amounting to 373,000, occurred in low- and middle-income countries (LMICs) that year, thereby highlighting a serious public health issue.

Persistent high-risk human papillomavirus (HPV) infection has been identified as the primary etiologic factor for cervical cancer [2]. It has been estimated that high-risk (HR) HPV types, namely HPV-16 and HPV-18, cause approximately 70% percent of cervical cancers [3]. Most HPV infections resolve spontaneously, but a small number of these infections will be persistent and lead to either pre-cancerous lesions or to the invasive form of cervical cancer [4].

Cervical cancer screening is one of the effective ways to prevent the development of precancerous cervical lesions and cervical cancer. Historically, cervical cancer prevention programs extensively employed the cytological examination of cervical epithelial cells via the Papanicolaou test (Pap smear, or Pap test) [5]. However, a widely introduced Pap test has a few drawbacks, which include low sensitivity/specificity and reliance on physician-collected samples [6]. Thus, the WHO suggests applying HPV DNA detection as a cervical cancer screening option starting at the age of 30 years with regular screening every 5 to 10 years [7]. HPV DNA testing is a novel alternative that is more sensitive due to the most recent developments and is becoming more available in numerous countries due to similar recent findings that show it is more predictive of precancerous lesions [8]. Nonetheless, screening rates are still not meeting the level of optimization, due to multiple factors/barriers such as lack of spouse or family support, cultural stigma around screening, lack of comfort with gynecological visits, and access to health care, especially in populations with lower-income and rural communities [9,10].

Self-collection methods have emerged primarily to address the challenges described above. One of the most common self-collection methods involves taking a vaginal swab or collecting a first-void urine sample. The non-invasive methods are gaining popularity as a valid alternative to clinician-collected cervical samples due to their excellent diagnostic accuracy [11]. Vaginal self-collection for HR-HPV testing is a viable method, with both diagnosis and treatment being equitably provided through clinic samples [11,12]. More recently, it has been postulated that urine testing may serve as a technically simple, non-invasive, and relatively low-cost method to increase mass screening [13]. First-void urine may hold additional promise since it provides a higher percentage of exfoliated cells emanating from the cervix and the vagina than does midstream urine [14]. Preliminary pilot data from India indicate that urine samples had a high degree of concordance with cervico-vaginal samples, with HR-HPV detection correctly identified by urine samples with 98.8% accuracy [4].

The purpose of this study is to conduct a systematic review and meta-analysis comparing the diagnostic accuracy of urine and vaginal self-sampling methods for the detection of high-risk HPV.

## 2. Materials and Methods

The systematic review and meta-analysis were conducted in accordance with the PRISMA 2020 (Preferred Reporting Items for Systematic Reviews and Meta-Analyses) guidelines. The review protocol was registered in the PROSPERO database (registration ID: CRD420251153008) to ensure transparency and reproducibility in accordance with PRISMA-P guidelines.

### 2.1. Search Strategy

A comprehensive electronic search was performed through major databases like PubMed, Scopus, Web of Science, and the Cochrane Library to identify relevant studies from January 2015 to October 2025. The search strategy employed Boolean operators “AND” and “OR”, Medical Subject Headings (MeSH), and free text words. Key MeSH terms included “Urine Specimen Collection,” “Papillomaviridae,” and “Vaginal Smears.” Free-text terms included “urine,” “first-void urine,” “vaginal self-sampling,” “vaginal swab,” “clinician-collected cervical sample,” “HPV,” “diagnostic accuracy,” “sensitivity,” and “specificity.” The complete search strategy for each database is presented in Supplementary Table S1. In addition to the database search, other relevant records were identified through manual checking of the reference lists of included studies and major reviews in the field. Detection of high-risk human papillomavirus was the main outcome under consideration, with the search of CIN2+ results whenever these had been provided.

### 2.2. Eligibility Criteria

Before the analysis of the data, all retrieved records were checked for accuracy, and duplicates were removed. Two reviewers independently assessed the titles and abstracts of the identified studies, followed by full-text reviews for eligibility determination. Disagreements were resolved by discussion among the authors.

The review included studies that satisfied the following criteria: (1) the diagnostic accuracy of HPV testing was assessed by using self-collected urine and/or vaginal samples; (2) clinician-collected samples (cytology, histology, or HPV testing) were the standard; (3) there was sufficient data to calculate sensitivity and specificity or to make a 2 × 2 contingency table; (4) the subjects were women eligible for cervical cancer screening; and (5) the article was published in English between 2015 and 2025. Only English-language studies were included for both methodological and practical reasons. Most high-quality, peer-reviewed studies relevant to this study are published in English, which helps to minimize the risk of missing critical evidence while ensuring that the results of the research can be replicated. Additionally, the process of translating articles into English requires a significant number of resources and can result in the misinterpretation of data. Using English as the only language also increases practical usability and allows for the consistent use of standardized tools for evaluating all the research included in this study.

The studies were excluded based on the following criteria: (1) there was no clear reference standard, or comparisons made with samples taken by the clinicians were not clear; (2) only acceptability was assessed, or the study was about an infection other than HPV; (3) were reviews, commentaries, case reports, or editorials that had no original data; (4) had no extractable data on the accuracy of the diagnosis; or (5) the research only included women with confirmed cervical cancer.

### 2.3. Data Extraction and Quality Assessment

Two independent reviewers followed a standardized template to extract data. Among the data collected were author, publication year, country, study design, sample size, participant description, index and reference tests, assay types, and diagnostic accuracy metrics, including sensitivity, specificity, predictive values, and agreement statistics. The reviewers had a consensus to resolve disagreements. Methodological quality and risk of bias were assessed using the Quality Assessment of Diagnostic Accuracy Studies-2 (QUADAS-2) tool [15]. Two independent reviewers conducted the evaluations, and a third reviewer intervened in case of any differences in opinion.

### 2.4. Data Synthesis and Analysis

Data were first cleaned and organized in Microsoft Excel (Version 16.109.3), while statistical modeling and quantitative synthesis were performed using Stata (version 19.5).

A bivariate random-effects model was applied to combine sensitivity and specificity results, which showed an interdependent relationship. A continuity correction of 0.5 was applied to studies containing zero cells where necessary. Outcome definitions differed across studies (e.g., CIN2+, CIN3+, HSIL/AIS, and HR-HPV positivity) and were harmonized into a CIN2+ equivalent outcome for pooled diagnostic accuracy analysis where applicable. Hierarchical Summary Receiver Operating Characteristic (HSROC) modeling and the Area Under the Curve (AUC) method were used to calculate the overall accuracy of their system. The I^2^ statistics showed moderate heterogeneity at values above 50% and high heterogeneity at values above 75%.

We applied univariate meta-regression analysis to logit-transformed data to determine how various factors, which included sampling modality, assay technology, geographic origin, and specimen storage protocols, affected the study outcomes.

To evaluate publication bias, we used Deeks’ funnel plot asymmetry test, where a *p*-value <0.01 was considered indicative of significant bias.

## 3. Results

### 3.1. Study Selection

The literature search identified a total of 316 records from four electronic databases: 202 from PubMed, 24 from the Cochrane Library, 66 from Web of Science, and 24 from Scopus. After the removal of 95 duplicate records, 221 articles were available for title and abstract screening. Of these, 170 were eventually excluded for not meeting the inclusion criteria. Specifically, 20 articles had no information on diagnostic accuracy, 38 were about the wrong study type or population, and 112 did not mention HPV testing or self-sampling.

A total of 51 full-text articles were sought for retrieval, and all 51 reports were successfully retrieved (*n* = 0 reports unavailable). Out of these 51 articles, 29 were excluded. The reasons for excluding these 29 articles include the following: 14 did not have a suitable index test or reference standard, 9 did not provide two-by-two contingency data that could be extracted, and 6 were excluded because of poor reporting, which made it difficult to do a meaningful synthesis.

**Figure 1:**
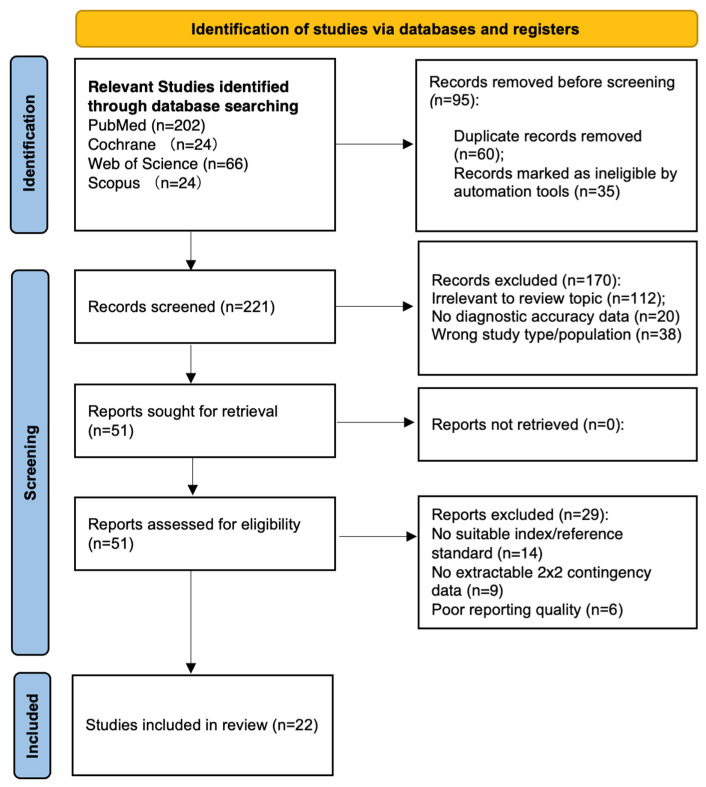
PRISMA flow diagram of systematic search and selection.

This screening process yielded a final pool of 22 eligible studies. To ensure our literature update was comprehensive, we cross-referenced our results with a recent meta-analysis by Li et al. (2025) [16]. While Li et al. originally included 15 studies, our independent database search successfully caught 14 of those foundational papers on its own, alongside 8 brand-new, more recent studies. The remaining 1 study from the Li et al. pool (published in 2014) was automatically excluded because our inclusion criteria restricted the timeline to 2015 onward. The PRISMA 2020 flowchart, which describes the study selection process, is shown in Figure 1.

### 3.2. General Description of the Included Studies

Table 1 shows the key features of the 22 studies that were included in the analysis [17,18,19,20,21,22,23,24,25,26,27,28,29,30,31,32,33,34,35,36,37,38]. During the period from 2015 to 2025, more than 9000 participants were involved in the research. Diagnostic accuracy studies carried out cross-sectional designs for all the studies, but there were two exceptions: one was a randomized trial for diagnostic accuracy [21] and the other paired diagnostic test accuracy study [22].

In the majority of studies, participants were women who were referred for colposcopy after having abnormal cytological results or receiving and reporting positive results on HPV screening. In a study [23], a small group of women with histologically confirmed CIN2+ lesions received special attention. The ages of the participants ranged from 17 to 85 years.

In every study, cervical specimens that were taken by a clinician were compared against at least one self-collected specimen (vaginal swab or urine sample). The collection tools used were Evalyn Brush^®^ (Rovers Medical Devices, Oss, The Netherlands) FLOQSwabs^®^ (Copan Diagnostics, Murrieta, CA, USA), Coari^®^ brushes (Kolplast Group, São Paulo, Brazil), Colli-Pee^®^ first-void urine collection devices (Novosanis NV, Wijnegem, Belgium), and standard sterile containers. The specimens were stored in a temperature range from room temperature to −80 °C.

A number of different commercial assays were used for the diagnosis of HPV. These included tests like CerviClear^®^, EUROArray HPV, CareHPV, Anyplex II HPV28, Abbott RealTime, BD Onclarity^®^, OncoPredict HPV QT/Screening, and Cobas^®^ 4800/8800. The type-specific results were available for HPV types 16 and 18, but the majority of tests were meant for the detection of the 14 high-risk HPV types only.

Histology (biopsy or excision), colposcopy-guided biopsy, or cervical cytology were among the reference standards that varied among studies. Based on the diagnostic results, there was a significant risk of HPV infection, or CIN2+ or CIN3+.

Sample sizes varied considerably from as few as 100 participants [23,24] to more than 2200 participants [25].

Among different studies, vaginal self-sampling consistently showed a higher agreement with clinician-collected samples than urine self-sampling, with kappa values often falling in the range of substantial to almost perfect (0.68–0.90). The sensitivity for self-collected urine samples ranged between 57.7% [26] and more than 90% [23]. Specificity for urine-based analysis was very high, often exceeding 90%.

### 3.3. Quality Assessment

The QUADAS-2 tool was used to evaluate the methodological quality of 22 research studies, as summarized in Table 1. Out of the 22 studies, patient selection was considered to have a low risk of bias in 17 (77.3%); in the rest of the studies, it was either medium or high due to being retrospective or lacking proper reporting. In most studies that did not disclose test intervals, the index test and reference standard domains were rated as low (20 of the 22 studies, 90.9%), while flow and timing were rated as low in 18 of the 22 studies (81.8%). Therefore, studies that did not indicate testing intervals were either medium or high risk.

In every sector, applicability concerns were generally minimal to medium. The reference standard had a low level of concern in 20 out of the 22 studies (90.9%), and the patient selection acceptability concerns were low in 18 out of 22 studies (81.8%). Out of 22 studies, 6 have been assigned a low level of concern for the index test (27.3%), while the remaining amount were rated at a medium level due to a lack of reporting on test interpretation. These findings support the overall methodological robustness and relevance of the included studies.

### 3.4. Pooled Diagnostic Accuracy of HPV Testing Across Sample Types

The meta-analysis included 22 studies and 53 diagnostic datasets (all individual cohort parameters, index molecular platforms, and raw 2 × 2 data matrices are detailed in Supplementary Table S2). Three comparison groups were analyzed: (1) vaginal self-sampling compared with clinician-collected cervical samples, (2) urine self-sampling versus clinician-collected cervical samples, and (3) direct head-to-head comparisons of vaginal and urine self-sampling. We used hierarchical summary receiver operating characteristic (HSROC) and bivariate random-effects models to calculate combined estimates of sensitivity and specificity.

**Figure 2:**
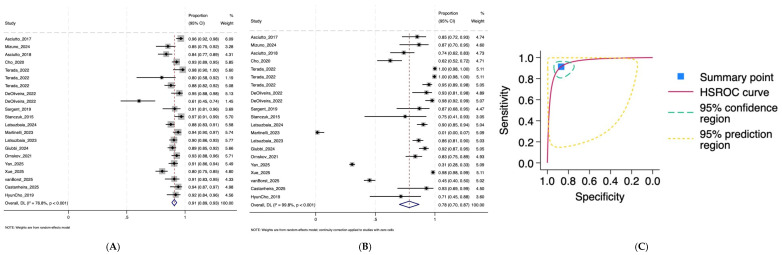
Diagnostic accuracy of vaginal self-sampling compared with clinician-collected cervical samples. (**A**) Forest plot of pooled sensitivity estimates; (**B**) Forest plot of pooled specificity estimates; (**C**) HSROC curve for CIN2+ detection. Footnote: Forest plots (**A**,**B**) illustrate individual study point estimates (squares, scaled by sample weight) and their 95% confidence intervals (horizontal lines) for the vaginal-to-clinician comparison; the final pooled meta-analytic estimates are represented by the diamonds. In the HSROC curve (**C**), the central summary point indicates the combined average sensitivity and specificity of vaginal self-sampling. The surrounding green dashed line defines the 95% confidence region, displaying the statistical precision of this pooled estimation. The outer yellow dashed line marks the 95% prediction region, representing the expected true performance variation in vaginal swabs across future, independent clinical settings [17,18,19,20,23,24,25,27,28,29,30,31,32,33,34,35,36,38].

Vaginal self-sampling showed high diagnostic accuracy across 21 different datasets. The pooled sensitivity for detecting CIN2+ lesions was 91.3% (95% CI: 84.1–95.4%), while the pooled specificity was 86.9% (95% CI: 78.4–92.4%) (Figure 2A,B). The pooled diagnostic odds ratio (DOR) was 69.9, indicating strong discriminatory ability in distinguishing CIN2+ cases from non-cases.

The HSROC plot (Figure 2C) visually supports these findings; the summary point is positioned in the upper-left quadrant, reflecting high performance. The tight 95% confidence region (green dashed line) indicates high precision in our estimates, while the 95% prediction region (yellow dashed line) shows that vaginal swabs maintain their high sensitivity across various study groups which display moderate differences.

**Figure 3:**
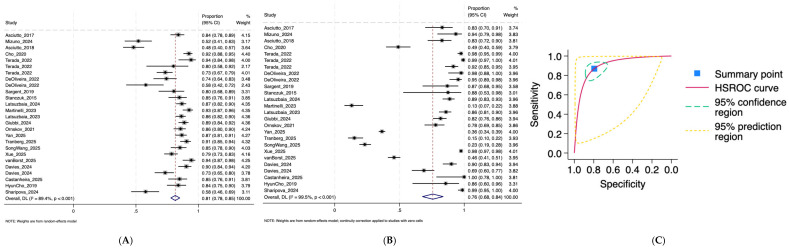
Diagnostic accuracy of urine self-sampling compared with clinician-collected cervical samples; (**A**) Forest plot of pooled sensitivity estimates; (**B**) Forest plot of pooled specificity estimates; (**C**) Hierarchical summary receiver operating characteristic (HSROC) curve for CIN2+ detection. Footnote: Forest plots (**A**,**B**) illustrate individual study point estimates (squares, scaled by sample weight) and their 95% confidence intervals (horizontal lines) for the urine-to-clinician comparison; the final pooled meta-analytic estimates are represented by the diamonds. In the HSROC curve (**C**), the central summary point indicates the combined average sensitivity and specificity of urine self-sampling. The surrounding green dashed line defines the 95% confidence region, displaying the statistical precision of this pooled estimation. The outer yellow dashed line marks the 95% prediction region, representing the expected true performance variation in urine sampling across future, independent clinical settings [17,18,19,20,21,22,23,24,25,26,27,28,29,30,31,32,33,34,35,36,37,38].

The analysis of 26 datasets comparing urine self-sampling with clinician-collected samples yielded a pooled sensitivity of 86.9% (95% CI: 78.2–92.5%) and a pooled specificity of 79.5% (95% CI: 69.5–86.8%) (Figure 3A,B). Urine sampling showed somewhat lower sensitivity than vaginal self-sampling, but specificity was consistently good.

The HSROC plot (Figure 3C) shows that individual study results produced different outcomes because of the wide 95% prediction region, which showed this study variability. Substantial heterogeneity was observed across studies, with I^2^ values of 89.4% for sensitivity and 99.5% for specificity. Given this extreme heterogeneity, especially when it comes to urine specificity (I^2^ =99.5%), these pooled estimates should be treated with strict caution as they represent an averaged performance across a wide set of very different study settings, rather than a uniform clinical expectation. The 95% confidence region indicates acceptable precision around the pooled estimate.

**Figure 4:**
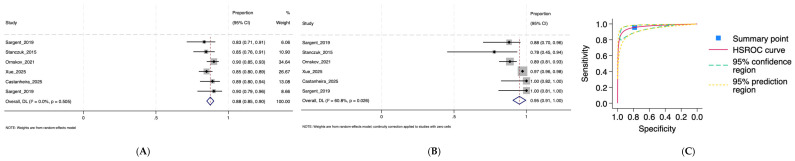
Diagnostic accuracy of urine self-sampling compared with vaginal self-sampling for CIN2+ detection. (**A**) Forest plot of pooled sensitivity estimates; (**B**) Forest plot of pooled specificity estimates; (**C**) Hierarchical summary receiver operating characteristic (HSROC) curve. Footnote: Forest plots (**A**,**B**) illustrate individual study point estimates (squares, scaled by sample weight) and their 95% confidence intervals (horizontal lines) for the urine-to-vaginal head-to-head comparison; the final pooled meta-analytic estimates are represented by the diamonds. In the HSROC curve (**C**), the central summary point indicates the combined averaged sensitivity and specificity of both self-sampling modalities relative to each other. The surrounding green dashed line defines the 95% confidence region, displaying the statistical precision of this pooled estimation. The outer yellow dashed line marks the 95% prediction region, representing the expected true performance variation between these two self-sampling methods across future, independent clinical settings [23,24,25,27,35].

Six datasets included direct head-to-head comparisons of urine and vaginal self-sampling techniques. Within these cohorts, the publication by Sargent et al. (2019) [27] contributed two distinct datasets because the authors evaluated two separate commercial assay platforms (Abbott RealTime and Roche Cobas) on the same group of participants. Vaginal self-sampling achieved a pooled sensitivity of 95.6% (95% CI: 89.8–98.1%) while maintaining a pooled specificity rate of 78.6% (95% CI: 53.2–92.2%) (Figure 4A,B). An HSROC plot (Figure 4C) shows accurate diagnostic results that include precise measurement results through its 95% confidence interval. The narrow 95% prediction region suggests a good performance rate for vaginal sampling within the scope of the assessed cohorts. However, because this analysis is limited to six independent datasets, the result provides supportive but limited evidence. Vaginal self-sampling demonstrated higher pooled sensitivity for CIN2+ detection compared with urine self-sampling within this limited comparative subset.

### 3.5. Meta-Regression and Heterogeneity Analysis

Meta-regression analysis was used to investigate whether differences in study characteristics, including continent, test type, HPV genotype group, storage conditions, and reference standard, were associated with variations in diagnostic performance. The comparison between vaginal self-sampling and clinician-collected data showed pooled sensitivity results that exceeded 76.8% of the total variability. The majority of study-level factors, including continent, storage conditions, assay type, and reference standard, showed no statistically significant association with sensitivity results. The HPV genotype distribution throughout the study population contributed to the observed study differences. The sensitivity of HPV16-specific studies showed a significant decrease when researchers measured high-risk HPV (β ≈ −3.48; *p* = 0.011). The HPV18 test results showed reduced sensitivity, but this finding did not reach statistical significance. Specificity analyses were less stable, and no consistent predictors of specificity were identified.

The comparison between urine self-sampling and clinician-collected samples showed different sensitivity results due to the presence of large study differences. According to meta-regression results, the assay type was associated with variation in diagnostic performance. The mRNA-based assays showed lower sensitivity results when compared to DNA-based platforms in these datasets (β ≈ −1.69; *p* = 0.024). The storage of samples at room temperature resulted in reduced sensitivity (*p* = 0.055), which showed an almost significant link to the storage conditions. Sensitivity showed no significant relationship with either geographic location, HPV genotype group, or reference standard. The specific analysis of study groups did not result in any stable predictive factors because of the combination of study variability and small subgroup sizes.

The direct vaginal and urine self-sampling methods showed minimal sensitivity differences, which resulted in consistent study results. The study included only 6 datasets, which reduced the statistical power of meta-regression analyses. The research failed to establish any reliable sensitivity predictors. European studies showed less specificity than studies from other regions, which created a potential geographic effect for specificity results (*p* ≈ 0.03). The research finding requires careful interpretation because of the limited number of studies and the unstable nature of subgroup models.

The meta-regression analysis showed that the diagnostic performance differences between tests were associated with two factors: assay technology and HPV genotype distribution. The continent and storage conditions, and reference standards, showed only limited effects on the majority of test comparisons.

### 3.6. Publication Bias

Publication bias was assessed using Deeks’ funnel plot asymmetry tests (Supplementary Figure S1A–C). The vaginal vs. clinician comparison showed significant asymmetry (*p* = 0.011, Supplementary Figure S1A), which occurred because outlier studies presented exceptionally high diagnostic odds ratios. The study found no significant bias in urine versus clinician (*p* = 0.191, Supplementary Figure S1B) or vaginal to urine direct comparison (*p* = 0.366, Supplementary Figure S1C) assessments. The results show that individual outliers exist, yet the combined urine and other testing methods give statistically valid results.

## 4. Discussion

Cervical cancer is a significant public health issue globally, most notably among lower- to middle-income nations with few screening programs [13]. HPV-based screening has the potential to significantly reduce incidence, but there are still numerous barriers to participation, including discomfort with pelvic exams, stigma, practical issues, and limited access to trained providers [12,39]. Alternative approaches to self-sampling, mostly vaginal or urinary, have recently gained traction in efforts to improve screening access and coverage [12]. Comparative diagnostic accuracy studies continue to demonstrate the effectiveness and efficacy of urine and vaginal self-sampling and to determine whether self-collected samples are as effective as clinician-collected samples [16]. This systematic review and meta-analysis update the evidence regarding the accuracy of self-sampling of urine and vaginal specimens for the detection of high-risk HPV, including evidence published since previous estimates were made. The intention is to broaden understanding of self-sampling performance and inform acceptability and implementation and assist in strategies for screening approaches and policy development.

The evidence we gathered from 22 research studies involving more than 9000 participants shows that vaginal self-sampling tests provide accurate results with 91.3% sensitivity and 86.9% specificity, which match the results from physician-collected samples. The self-collected urine samples achieved an overall test performance that showed 86.9% sensitivity and 79.5% specificity. Urine sampling produced more false negative results compared to vaginal sampling, but it still serves as a clinically useful testing method. Our head-to-head direct study comparison showed that vaginal swabs achieved a 95.6% testing accuracy, which they maintained with complete consistency (I^2^ = 0.0%). These findings support the broader implementation of both testing methods, but vaginal self-sampling serves as the superior method for detecting CIN2+ lesions.

Our research used meta-regression analysis to determine specific factors that impact diagnostic performance. The study discovered that assay technology serves as a significant contributor to testing results because mRNA-based assays showed a significant reduction in detection capabilities when compared to DNA-based PCR tests (β ≈ −1.69; *p* = 0.024). The data show that high-performance PCR platforms such as Cobas 4800/8800 and Alinity m provide better test results than mRNA or antigen-based tests such as careHPV [16]. Through our research, we discovered that testing for HPV16 alone resulted in a major decrease in sensitivity (*p* = 0.011) when compared to using pooled HR-HPV panels. The current data demonstrate that broad DNA-PCR testing must take priority for achieving maximum sensitivity because urine tests have shown comparable detection power to clinician samples for HPV 16/18 [40,41].

The research showed that participant satisfaction levels remained high, with more than 95% of study participants from 12 studies assessing self-sampling as highly acceptable. Both methods decreased shame and discomfort, which people experience when physicians take their samples, as previous research demonstrated that these methods serve as effective screening solutions for populations who lack screening access [4,42,43]. However, our findings suggest that urine-based testing methods are more “context-sensitive” than vaginal swab tests. Specifically, our analysis found a nearly significant association between urine sample storage at room temperature and decreased sensitivity, which requires standardized collection and transport methods to achieve dependable results.

The review demonstrates its main strength through the inclusion of 2024–2025 data and its implementation of meta-regression, which shows assay technology and HPV genotypes as the primary factors that create research heterogeneity.

The study presents multiple challenges that require resolution. The majority of studies examined high-risk referred populations, which restrict their findings to apply only to general screening environments. Our research discovered clear predictors of sensitivity, which the limited head-to-head datasets (*n* = 6) and extreme urine specificity variation (I^2^ = 99.5%) prevented us from establishing reliable subgroup models. Outcome definitions varied across studies (e.g., CIN2+, CIN3+, HSIL/AIS, and HR-HPV positivity), which reflects a varied composite reference standard across the literature and may have contributed to heterogeneity in pooled estimates. The absence of standardized urine collection procedures, which include first-void timing across studies, leads to the widespread study results showing high variability.

The practical implications of this study results are considerable. The availability of self-sampling methods may narrow the gap, enhance cervical cancer screening rates, and also increase participation among individuals who are reluctant to have cervical testing completed in an office. These approaches are particularly important for countries with low/insufficient coverage of cervical cancer screening and a high proportion of women positive for high-risk HPV [44,45,46]. Because self-sampling protocols are highly acceptable to patients, they represent an ideal strategy to bridge these coverage gaps in areas where routine clinical screening remains limited. Urine self-collection can be a beneficial option when patient concerns are primarily centered on privacy, acceptability, and accessibility; however, vaginal self-collection remains the preferred primary recommendation when maximizing clinical sensitivity is the main objective. The research results show that mRNA technology and improper storage methods decrease diagnostic accuracy, which needs to be combined with high-quality DNA-PCR testing methods for successful policy execution. The implementation of self-sampling techniques into national screening programs will enable faster detection of HPV infections, which will lead to a major reduction in the worldwide incidence of cervical cancer.

## 5. Conclusions

The study results prove that both vaginal self-collection and urine self-collection serve as effective tools for testing purposes. Vaginal self-sampling achieves diagnostic results that closely match those obtained from clinician-collected samples, while first-void urine self-sampling remains a non-invasive alternative. Despite being based on a limited number of datasets, the direct comparisons indicate that vaginal self-sampling has higher clinical sensitivity than urine self-sampling for CIN2+ detection, highlighting the need for suitably powered head-to-head trials in the future. Both methods show their highest effectiveness when laboratories use advanced DNA-based PCR testing due to better stability and sensitivity than mRNA-based testing across various sample types. These findings support the integration of self-sampling methods into existing cervical cancer screening programs, particularly for under-screened populations.

## Figures and Tables

**Table 1:** Key characteristics of the included studies.

	Author (Year)	Country	Setting/Recruitment	Study Design	Sample Size	Sample Types Compared	Assay	Reference Standard	Outcome	Overall Quality	Key Notes
1	Asciutto (2017) [17]	Sweden	Colposcopy clinic	Cross-sectional	218	Urine, vaginal, cervical	Cobas 4800	Colposcopy + biopsy	HSIL/AIS	Medium	Vaginal self-sampling is nearly as sensitive as cervical; urine is slightly less; Cobas 4800 is reliable; good agreement with biopsy.
2	Mizuno (2024) [18]	Japan	Hospital	Cross-sectional	121	Urine, vaginal, cervical	BD Onclarity	Cervical cytology	CIN2+	Medium	Vaginal and urine are comparable to cervical cytology; supports self-sampling in hospital settings.
3	Asciutto (2018) [19]	Sweden	Clinic	Cross-sectional	209	Urine, vaginal, cervical	Aptima mRNA	Colposcopy + biopsy	HSIL/AIS/cancer	Medium	Vaginal self-samples are accurate; urine is less sensitive; high concordance with clinician sampling.
4	Cho (2021) [20]	Korea	Colposcopy center	Cross-sectional	314	Urine, vaginal, cervical	Anyplex II; RealTime HR S	Colposcopy + biopsy	CIN2+/CIN3+	Medium	Urine and vaginal are comparable to cervical; reliable assays; colposcopy referral population.
5	Tranberg (2025) [21]	Denmark	Colposcopy/LEEP referral	Paired diagnostic test accuracy	325	First-void urine vs. cervical (±vaginal)	Allplex HR HPV DNA	Histology	CIN2+/CIN3+	Low	First-void urine comparable to cervical; paired diagnostic design.
6	Davies (2024) [22]	UK	Colposcopy clinic	Randomized diagnostic accuracy trial	480 (465 pairs)	FVU vs. cervical; standard pot vs. cervical	Roche Cobas 8800	Colposcopy ± histology	CIN2+/CIN3+	Low	Randomized DTA trial; urine feasible; high CIN2+/CIN3+ detection.
7	Castanheira (2025) [23]	Brazil	Tertiary hospital	Cross-sectional	100	Urine, vaginal, cervical	Cobas 4800	Histology	CIN2+/CIN3+	Low	Vaginal and urine reliable; Cobas 4800 effective; small tertiary study.
8	Stanczuk (2015) [24]	UK	Clinic	Cross-sectional	100	Urine, vaginal, cervical	Cobas 4800	Cervical cytology	CIN2+/CIN3+	Medium	Vaginal comparable to cervical; urine slightly lower; Cobas 4800 reliable.
9	Xue (2025) [25]	China	Hospital	Cross-sectional	2228	Urine, vaginal, cervical	CareHPV; HBRT-H14	Histology	CIN2+	Medium	Large hospital cohort; vaginal and urine feasible; effective assays.
10	Sharipova (2024) [26]	Uzbekistan	Hospital	Cross-sectional (with control group)	218	Urine vs. cervical	AmpliSens PCR	Cervical smear PCR	HR-HPV positivity		Urine vs. cervical; AmpliSens PCR accurate; includes control group.
11	Sargent (2019) [27]	UK	Colposcopy clinic	Cross-sectional	104	Urine, vaginal, cervical	Abbott RealTime; Roche Cobas	Cervical cytology	CIN2+	Medium	Self-samples comparable to cervical; valid assays; small UK study.
12	Terada (2022) [28]	Japan	Hospital	Cross-sectional	300	Urine, vaginal, cervical	Cobas 8800	Cervical cytology	CIN2+/CIN3+/microinvasive	Medium	Vaginal slightly more sensitive; Cobas 8800 detected microinvasive lesions.
13	Oliveira (2020) [29]	Brazil	Clinic	Cross-sectional	124	Urine, vaginal, cervical	Cobas 4800; OncoE6	Histology	CIN2/3	High	Vaginal accurate; urine lower sensitivity; histology confirmed outcomes.
14	Latsuzbaia (2024) [30]	Italy	Referral clinic	Cross-sectional	463	Urine, vaginal, cervical	OncoPredict HPV QT	Colposcopy + biopsy	CIN2+	Medium	Vaginal higher sensitivity; large referral clinic; good concordance with biopsy.
15	Martinelli (2023) [31]	Italy	Clinic	Cross-sectional	245	Urine, vaginal, cervical	Anyplex II HPV28	Colposcopy + biopsy	CIN2+	Medium	Vaginal more sensitive; Anyplex II accurate; clinic setting.
16	Latsuzbaia (2023) [32]	Belgium	Clinic	Cross-sectional	499	Urine, vaginal, cervical	Alinity m HR HPV	HPV infection/cytology	CIN2+	High	Alinity m reliable; vaginal > urine; high concordance.
17	Giubbi (2024) [33]	Italy	Colposcopy centers	Cross-sectional	490	Urine, vaginal, cervical	OncoPredict Screening	HPV infection/cytology	CIN2+	Medium	Vaginal and urine effective; moderate-to-high agreement.
18	Cho (2019) [34]	Korea	Medical centers	Cross-sectional	101	Urine, vaginal, cervical	RealTime HR S; Anyplex II	Clinician sampling	HPV positive	Medium	Urine and vaginal accurate; assays reliable.
19	Ornskov (2021) [35]	Denmark	Clinic	Cross-sectional	305	Urine, vaginal, cervical	Cobas	Histology	CIN2+/CIN3+	Medium	Vaginal slightly better; Cobas reliable; histology reference.
20	Yan (2025) [36]	China	Colposcopy clinics	Prospective cross-sectional (DTA)	1588	Urine, vaginal, cervical	CerviClear HR-HPV PCR	Histology	CIN2+/CIN3+	Medium	Large prospective study; both self-samples, accurate, reliable assay.
21	Song & Wang (2025) [37]	China	Hospital	Cross-sectional	458	Urine vs. cervical	PCR-based HR-HPV panel	Histology	CIN2+	Medium	Urine has slightly lower sensitivity but high specificity.
22	van den Borst (2025) [38]	Belgium	Colposcopy clinics (VALHUDES)	Cross-sectional	499	First-void urine, vaginal, cervical	EUROArray HPV	Colposcopy ± biopsy	CIN2+/CIN3+	Low	VALHUDES study; first-void urine is accurate; vaginal slightly higher sensitivity.

## Data Availability

Not applicable.

## Supplementary Materials

The following supporting information can be downloaded at: https://www.mdpi.com/article/10.3390/v18060681/s1, Table S1: Search Strategies by Database (January 2015–October 2025); Figure S1: Deeks’ funnel plot assessing publication bias for HR-HPV detection: (A) vaginal versus clinician-collected samples, (B) urine versus clinician-collected samples, and (C) vaginal versus urine self-sampling; Table S2: Comprehensive extraction matrix of the 53 diagnostic datasets from the 22 included studies, detailing baseline demographics, molecular assay specifications, raw 2 × 2 contingency outcomes (TP, FP, FN, TN), and specific composite reference standard target conditions; File S1: PRISMA 2020 checklist; File S2; PRISMA 2020 abstract checklist [47].

## Abbreviations

The following abbreviations are used in this manuscript:

AUC	Area Under the Curve
CIN	Cervical Intraepithelial Neoplasia
CIN2+	Cervical Intraepithelial Neoplasia grade 2 or higher
CIN3+	Cervical Intraepithelial Neoplasia grade 3 or higher
DOR	Diagnostic Odds Ratio
DNA	Deoxyribonucleic Acid
EUROArray	EUROArray HPV test system
HPV	Human Papillomavirus
HSIL	High-Grade Squamous Intraepithelial Lesion
HSROC	Hierarchical Summary Receiver Operating Characteristic
HR-HPV	High-Risk Human Papillomavirus
LEEP	Loop Electrosurgical Excision Procedure
LMICs	Low- and Middle-Income Countries
mRNA	Messenger Ribonucleic Acid
PCR	Polymerase Chain Reaction
QUADAS-2	Quality Assessment of Diagnostic Accuracy Studies-2
ROC	Receiver Operating Characteristic
WHO	World Health Organization
