# Supplementary material for: Diagnostic Accuracy of Urine and Vaginal Self-Sampling for Detection of High-Risk Human Papillomavirus: A Systematic Review and Meta-Analysis
Source: Viruses. 2026 Jun 18;18(6):681. doi: 10.3390/v18060681 (PMC13308321; doi:10.3390/v18060681)
Supplement: Supplementary file 1 [file viruses-18-00681-s001.zip › Supplementary Figure S1.pdf]

Supplementary Figure S1.

Deeks' funnel plot assessing publication bias for hrHPV detection: (A) vaginal versus clinician-collected samples, (B) urine versus clinician-collected samples, and (C) vaginal versus urine self-sampling.

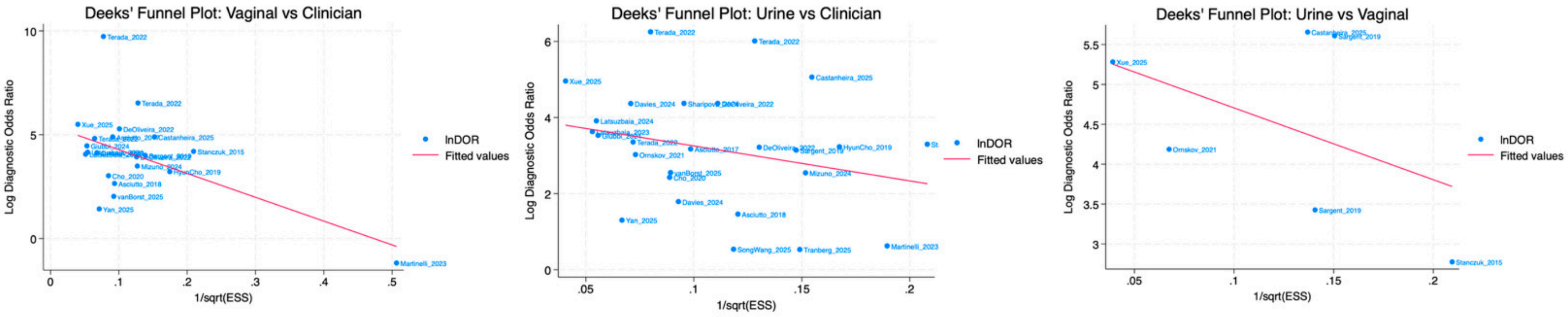

(A) (B) (C)
