# Supplementary material for: Diagnostic Accuracy of Urine and Vaginal Self-Sampling for Detection of High-Risk Human Papillomavirus: A Systematic Review and Meta-Analysis
Source: Viruses. 2026 Jun 18;18(6):681. doi: 10.3390/v18060681 (PMC13308321; doi:10.3390/v18060681)
Supplement: Supplementary file 1 [file viruses-18-00681-s001.zip › Supplementary Table S1.pdf]

Supplementary Table S1. Search Strategies by Database (January 2015–October 2025)

Database	Search Strategy	Records Retrieved
**PubMed**	#1 ("urine sample"[tiab] OR "urine self"[tiab] OR "urine self-collection"[tiab] OR "urine specimen"[tiab] OR "first-void urine"[tiab] OR "Urine Specimen Collection"[Mesh])<br>#2 ("human papillomavirus"[Mesh] OR HPV[tiab] OR "human papillomavirus"[tiab])<br>#3 ("vaginal self-sample"[tiab] OR "vaginal sample"[tiab] OR "vaginal self-collection"[tiab] OR "vaginal swab"[tiab] OR "vaginal specimen"[tiab] OR "Vaginal Smears"[Mesh])<br>#4 ("sensitivity"[tiab] OR "specificity"[tiab] OR "accuracy"[tiab] OR "agreement"[tiab] OR "concordance"[tiab] OR "diagnostic"[tiab])<br>#5 #1 AND #2 AND #3 AND #4	202
**Scopus**	TITLE-ABS-KEY ("human papillomavirus" OR HPV OR "high-risk HPV" OR "HPV DNA") AND TITLE-ABS-KEY ("urine sample" OR "urine self-sample" OR "urine self-collection" OR "first-void urine") AND TITLE-ABS-KEY ("vaginal sample" OR "vaginal self-sample" OR "vaginal self-collection" OR "vaginal swab") AND TITLE-ABS-KEY ("cervical sample" OR "cervical screening" OR "cervical intraepithelial neoplasia" OR CIN) AND TITLE-ABS-KEY (sensitivity OR specificity OR "diagnostic accuracy" OR "positive predictive value" OR "negative predictive value" OR DOR OR "likelihood ratio" OR "ROC curve" OR "area under curve")	24
**Cochrane Library**	#1 (urine OR "urine self-sample" OR "urine self-collection" OR "first-void urine"):ti,ab,kw<br>#2 ("human papillomavirus" OR HPV OR "high-risk HPV" OR "HPV DNA"):ti,ab,kw<br>#3 (vaginal OR "vaginal self-sample" OR "vaginal self-collection" OR "vaginal swab"):ti,ab,kw<br>#4 (cervical OR "cervical cancer screening" OR "cervical intraepithelial neoplasia" OR CIN):ti,ab,kw<br>#5 #1 AND #2 AND #3 AND #4	24
**Web of Science**	TS=("human papillomavirus" OR HPV OR "high-risk HPV" OR "HPV DNA") AND TS=(urine OR "urine sample" OR "first-void urine") AND TS=(vaginal OR "vaginal sample" OR "vaginal swab") AND TS=(cervical OR CIN OR "cervical screening") AND TS=(accuracy OR sensitivity OR specificity OR "test performance" OR "diagnostic value" OR DOR OR "likelihood ratio")	66
