# Supplementary material for: Diagnostic Accuracy of Urine and Vaginal Self-Sampling for Detection of High-Risk Human Papillomavirus: A Systematic Review and Meta-Analysis
Source: Viruses. 2026 Jun 18;18(6):681. doi: 10.3390/v18060681 (PMC13308321; doi:10.3390/v18060681)
Supplement: Supplementary file 1 [file viruses-18-00681-s001.zip › Supplementary Table S2.pdf]

Supplementary Table S2.: Comprehensive extraction matrix of the 53 diagnostic datasets from the 22 included studies, detailing baseline demographics, molecular assay specifications, raw contingency outcomes (TP, FP, TN, FN), and specific composite reference standard target conditions.

№	Study ID (Author, Year)	Country	Cohort Size (N) & Target Population	Sampling Comparison (Index Test vs. Comparator)	Assay Platform & Target	HPV Subtype	TP	FP	FN	TN	Reference Standard & Target Condition
1	Asciutto 2017 [17]	Sweden	N=213; Colposcopy Referral	VSS vs. CCS	Cobas 4800 (DNA)	hrHPV (All)	160	7	6	40	Histology (CIN2+ / HSIL)
2	Asciutto 2017 [17]	Sweden	N=215; Colposcopy Referral	FVU vs. CCS	Cobas 4800 (DNA)	hrHPV (All)	141	8	27	39	Histology (CIN2+ / HSIL)
3	Mizuno 2024 [18]	Japan	N=98; Screening Cohort	VSS vs. CCS	BD Onclarity (DNA)	hrHPV (All)	58	4	10	26	Cytology (CIN2+)
4	Mizuno 2024 [18]	Japan	N=108; Screening Cohort	FVU vs. CCS	BD Onclarity (DNA)	hrHPV (All)	40	2	37	29	Cytology (CIN2+)
5	Asciutto 2018 [19]	Sweden	N=205; Colposcopy Referral	VSS vs. CCS	Aptima (mRNA)	hrHPV (All)	114	18	22	51	Histology (CIN2+ / Cancer)
6	Asciutto 2018 [19]	Sweden	N=195; Colposcopy Referral	FVU vs. CCS	Aptima (mRNA)	hrHPV (All)	63	11	68	53	Histology (CIN2+ / Cancer)
7	Cho 2021 [20]	Korea	N=334; Gynecology Clinic	VSS vs. CCS	Realtime HR-S (DNA)	hrHPV (All)	217	30	17	50	Histology (CIN2+ / CIN3+)
8	Cho 2021 [20]	Korea	N=314; Gynecology Clinic	FVU vs. CCS	Realtime HR-S (DNA)	hrHPV (All)	194	53	16	51	Histology (CIN2+ / CIN3+)
9	Terada 2022 [28]	Japan	N=300; Outpatient Clinic	VSS vs. CCS	Cobas 8800 (DNA)	HPV16	50	0	1	249	Histology (CIN2+ / CIN3+)
10	Terada 2022 [28]	Japan	N=300; Outpatient Clinic	FVU vs. CCS	Cobas 8800 (DNA)	HPV16	48	6	3	243	Histology (CIN2+ / CIN3+)
11	Terada 2022 [28]	Japan	N=300; Outpatient Clinic	VSS vs. CCS	Cobas 8800 (DNA)	HPV18	16	1	4	279	Cytology (CIN2+ / CIN3+)

**12**	Terada 2022 [28]	Japan	N=300; Outpatient Clinic	FVU vs. CCS	Cobas 8800 (DNA)	HPV18	16	2	4	278	Cytology (CIN2+ / CIN3+)
**13**	Terada 2022 [28]	Japan	N=300; Outpatient Clinic	VSS vs. CCS	Cobas 8800 (DNA)	Other hrHPV	159	6	22	113	Cytology (CIN2+ / CIN3+)
**14**	Terada 2022 [28]	Japan	N=300; Outpatient Clinic	FVU vs. CCS	Cobas 8800 (DNA)	Other hrHPV	133	10	48	109	Cytology (CIN2+ / CIN3+)
**15**	Oliveira 2020 [29]	Brazil	N=124; HIV+ Screened Cohort	VSS vs. CCS	Cobas 4800 (DNA)	hrHPV (All)	78	3	4	39	Cytology (CIN2+ / 3+)
**16**	Oliveira 2020 [29]	Brazil	N=125; HIV+ Screened Cohort	FVU vs. CCS	Cobas 4800 (DNA)	hrHPV (All)	61	12	11	41	Cytology (CIN2+ / 3+)
**17**	Oliveira 2020 [29]	Brazil	N=124; HIV+ Screened Cohort	VSS vs. CCS	OncoE6 (Protein)	HPV16/18-E6	23	2	15	84	Histology (CIN2+ / 3+)
**18**	Oliveira 2020 [29]	Brazil	N=124; HIV+ Screened Cohort	FVU vs. CCS	OncoE6 (Protein)	HPV16/18-E6	22	4	16	82	Histology (CIN2+ / 3+)
**19**	Sargent 2019 [27]	UK	N=79; Colposcopy Clinic	VSS vs. CCS	Abbott RT (Protein)	hrHPV (All)	51	3	5	20	Histology (CIN2+)
**20**	Sargent 2019 [27]	UK	N=79; Colposcopy Clinic	FVU vs. CCS	Abbott RT (Protein)	hrHPV (All)	45	3	11	20	Histology (CIN2+)
**21**	Sargent 2019 [27]	UK	N=79; Colposcopy Clinic	Head-to-head comparison (VSS vs. FVU)	Abbott RT (DNA)	hrHPV (All)	45	3	9	22	Cytology (CIN2+)
**22**	Stanczuk 2015 [24]	UK	N=100; Screen-Positive Cohort	VSS vs. CCS	Cobas 4800 (DNA)	hrHPV (All)	89	2	3	6	Cytology (CIN2+ / CIN3+)
**23**	Stanczuk 2015 [24]	UK	N=100; Screen-Positive Cohort	FVU vs. CCS	Cobas 4800 (DNA)	hrHPV (All)	78	1	14	7	Cytology (CIN2+ / CIN3+)
**24**	Stanczuk 2015 [24]	UK	N=100; Screen-Positive Cohort	Head-to-head comparison	Cobas 4800 (DNA)	hrHPV (All)	77	2	14	7	Cytology (CIN2+ / CIN3+)

				(VSS vs. FVU)							
**25**	Latsuzbaia 2024 [30]	Italy	N=452; Routine Screening	VSS vs. CCS	OncoPredict QT (DNA)	hrHPV (All)	244	17	34	157	Cytology (CIN2+)
**26**	Latsuzbaia 2024 [30]	Italy	N=450; Routine Screening	FVU vs. CCS	OncoPredict QT (DNA)	hrHPV (All)	236	20	36	158	Cytology (CIN2+)
**27**	Martinelli 2023 [31]	Italy	N=272; Outpatient Screening	VSS vs. CCS	Anyplex II (DNA)	hrHPV (All)	160	7	4	101	Cytology (CIN2+)
**28**	Martinelli 2023 [31]	Italy	N=279; Outpatient Screening	FVU vs. CCS	Anyplex II (DNA)	hrHPV (All)	149	7	12	111	Cytology (CIN2+)
**29**	Latsuzbaia 2023 [32]	Italy	N=492; Clinic-Based Cohort	VSS vs. CCS	Alinity m (DNA)	hrHPV (All)	246	30	26	190	Cytology (CIN2+)
**30**	Latsuzbaia 2023 [32]	Italy	N=492; Clinic-Based Cohort	FVU vs. CCS	Alinity m (DNA)	hrHPV (All)	247	29	39	177	Histology (CIN2+)
**31**	Giubbi 2024 [33]	Italy	N=449; Screening Cohort	VSS vs. CCS	OncoPredict (DNA)	hrHPV (All)	241	15	29	164	Histology (CIN2+)
**32**	Giubbi 2024 [33]	Italy	N=447; Screening Cohort	FVU vs. CCS	OncoPredict (DNA)	hrHPV (All)	212	38	27	170	Histology (CIN2+)
**33**	Ornskov 2021 [35]	Denmark	N=305; Non-Attender Program	VSS vs. CCS	Cobas 4800 (DNA)	hrHPV (All)	182	18	14	91	Histology (CIN2+ / CIN3+)
**34**	Ornskov 2021 [35]	Denmark	N=305; Non-Attender Program	FVU vs. CCS	Cobas 4800 (DNA)	hrHPV (All)	168	24	28	85	Composite (Clinician Consensus)
**35**	Ornskov 2021 [35]	Denmark	N=305; Non-Attender Program	Head-to-head comparison (VSS vs. FVU)	Cobas 4800 (DNA)	hrHPV (All)	180	12	20	93	Composite (Clinician Consensus)
**36**	Yan 2025 [36]	China	N=1243; Community Screen	VSS vs. CCS	CerviClear (DNA)	hrHPV (All)	184	96	21	942	Composite (Clinician Consensus)
**37**	Yan 2025 [36]	China	N=539; Community Screen	FVU vs. CCS	CerviClear (DNA)	hrHPV (All)	176	88	0	275	Composite (Clinician Consensus)

**38**	Tranberg 2025 [21]	Denmark	N=928; Home-Based Cohort	FVU vs. CCS	Allplex HR (DNA)	hrHPV (All)	163	12	31	722	Composite (Clinician Consensus)
**39**	SongWang 2025 [37]	China	N=324; Rural Screening	FVU vs. CCS	PCR-based (DNA)	hrHPV (All)	104	25	8	187	Composite (Clinician Consensus)
**40**	Xue 2025 [25]	China	N=2210; Population Trial	VSS vs. CCS	CareHPV (DNA)	hrHPV (All)	176	32	43	1959	Histology (CIN2+)
**41**	Xue 2025 [25]	China	N=2213; Population Trial	FVU vs. CCS	CareHPV (DNA)	hrHPV (All)	172	49	47	1945	Histology (CIN2+)
**42**	Xue 2025 [25]	China	N=2200; Population Trial	Head-to-head comparison (VSS vs. FVU)	CareHPV (DNA)	hrHPV (All)	186	54	33	1927	Histology (CIN2+)
**43**	Van den Borst 2025 [38]	Belgium	N=311; Colposcopy Referral	VSS vs. CCS	EUROArray (DNA)	hrHPV (All)	78	22	28	183	Histology (CIN2+ / CIN3+)
**44**	Van den Borst 2025 [38]	Belgium	N=296; Colposcopy Referral	FVU vs. CCS	EUROArray (DNA)	hrHPV (All)	83	22	5	186	Histology (CIN2+ / CIN3+)
**45**	Davies 2024 [22]	UK	N=245; Clinic Validation	FVU (Colli-Pee) vs. CCS	Cobas 8800 (DNA)	hrHPV (All)	112	12	12	109	Histology (CIN2+ / CIN3+)
**46**	Davies 2024 [22]	UK	N=230; Clinic Validation	FVU (Standard Cup)vs. CCS	Cobas 8800 (DNA)	hrHPV (All)	91	33	33	73	Histology (CIN2+ / CIN3+)
**47**	Castanheira 2025 [23]	Brazil	N=100; Low-Income Screening	VSS vs. CCS	Cobas 4800 (DNA)	hrHPV (All)	81	1	5	13	Histology (CIN2+ / CIN3+)
**48**	Castanheira 2025 [23]	Brazil	N=100; Low-Income Screening	FVU vs. CCS	Cobas 4800 (DNA)	hrHPV (All)	73	0	13	14	Histology (CIN2+ / CIN3+)
**49**	Castanheira 2025 [23]	Brazil	N=100; Low-Income Screening	Head-to-head comparison (VSS vs. FVU)	Cobas 4800 (DNA)	hrHPV (All)	73	0	9	18	Histology (CIN2+ / CIN3+)
**50**	HyunCho 2019 [34]	Korea	N=101; Follow-up Cohort	VSS vs. CCS	Realtime HR-S (DNA)	hrHPV (All)	80	4	7	10	Histology (HPV+ Baseline)

**51**	HyunCho 2019 [34]	Korea	N=101; Follow-up Cohort	FVU vs. CCS	Realtime HR-S (DNA)	hrHPV (All)	73	2	14	12	Histology (HPV+ Baseline)
**52**	Sharipova 2024 [26]	Uzbekistan	N=218; Regional Screen	FVU vs. CCS	AmpliSens (DNA)	hrHPV (All)	41	2	30	145	Composite (Clinician Consensus)
**53**	Sargent 2019 [27]	UK	N=66; Colposcopy Clinic	Head-to-head comparison (VSS vs. FVU)	Cobas 4800 (DNA)	hrHPV (All)	45	0	5	16	Cytology (CIN2+)

***Abbreviations:**

VSS: Vaginal Self-Sample

FVU: First-Void Urine

CCS: Clinician-Collected Sample

hrHPV: High-risk Human Papillomavirus

TP / FP / FN / TN: True Positive / False Positive / False Negative / True Negative

CIN2+ / CIN3+: Cervical Intraepithelial Neoplasia Grade 2 / 3 or worse

HSIL: High-Grade Squamous Intraepithelial Lesion

AIS / Micro CA: Adenocarcinoma in situ / Microinvasive Carcinoma
